# A Novel Nanofabrication Technique of Silicon-Based Nanostructures

**DOI:** 10.1186/s11671-016-1702-4

**Published:** 2016-11-15

**Authors:** Lingkuan Meng, Xiaobin He, Jianfeng Gao, Junjie Li, Yayi Wei, Jiang Yan

**Affiliations:** 1 Key Laboratory of Microelectronics Devices and Integrated Technology, Institute of Microelectronics of Chinese Academy of Sciences, Beijing, 100029 People’s Republic of China; 2 University of Chinese Academy of Sciences, Beijing, 100049 People’s Republic of China

**Keywords:** Nanofabrication technique, Amorphous silicon, Silicon-based nanostructures, Nanotrench, Nanoline, Nanofin, 81.16.Rf

## Abstract

A novel nanofabrication technique which can produce highly controlled silicon-based nanostructures in wafer scale has been proposed using a simple amorphous silicon (α-Si) material as an etch mask. SiO_2_ nanostructures directly fabricated can serve as nanotemplates to transfer into the underlying substrates such as silicon, germanium, transistor gate, or other dielectric materials to form electrically functional nanostructures and devices. In this paper, two typical silicon-based nanostructures such as nanoline and nanofin have been successfully fabricated by this technique, demonstrating excellent etch performance. In addition, silicon nanostructures fabricated above can be further trimmed to less than 10 nm by combing with assisted post-treatment methods. The novel nanofabrication technique will be expected a new emerging technology with low process complexity and good compatibility with existing silicon integrated circuit and is an important step towards the easy fabrication of a wide variety of nanoelectronics, biosensors, and optoelectronic devices.

## Background

Silicon-based nanostructures including Si and SiO_2_ are of great technological importance and considerable interest, and they have been extensively exploited for a wide variety of scientific and engineering applications ranging from attractive plasmonic [1, 2], sensitive biosensor devices [3, 4], phonics crystals to magnetic storage media [5, 6] and are also the building blocks for a broad range of nanoelectronic devices such as MOSFET [7, 8], nanofluidics [9, 10], and optoelectronics devices [11, 12]. Accordingly, there have been a large number of fabrication techniques and methods to produce highly controlled silicon-based nanostructures using top-down or bottom-up patterning strategies in the literatures [13–15]. Based on these lithographic features, to fabricate successfully nanostructures, it is easily understood that a most significant point is in achieving a good pattern transfer with a high fidelity into the underlying substrate materials. Although a large number of techniques capable of fabricating nanofeatures have been developed and are well understood, including conventional photolithography, electron beam lithography, nanosphere lithography, nanoimprint lithography, and self-assembly of block copolymer, these techniques have encountered their own significant challenges when patterning nanoscale features for some specific applications.

In addition, it is well known that fabrication of SiO_2_ nanostructures with a large scale such as micrometer or sub-micrometer is fairly easily obtained by using a simple photo resist (PR) as an etch mask. However, as critical dimension (CD) continuously scales below 100 nm, some critical requirements, such as accurate control of CD, line edge roughness (LER), and etch uniformity, have been becoming more and more challenging [16, 17]. In this case, it is very difficult for PR as a direct etch mask to feature various nanostructures due to poor plasma etching resistances and thin PR thickness. For example, for typical top-down fabrication such as 193-nm lithography or electron beam lithography, the thickness of PR is required to be as thin as possible in order to improve lithography resolution and at the same time to avoid the pattern collapse of PR [18]. Accordingly, such thin PR thickness in turn limits the process capability of dry etcher and may bring a lot of problems including accurate size control, LER, and etch uniformity when the PR patterns are transferred to underlying substrate materials. In this case, it will be very difficult to realize a good patterning by only PR as an etch mask, as revealed previously by us.

Actually, it is particularly difficult to fabricate well-controlled SiO_2_ nanostructures with a good regularity and controllability in pattern size, edge roughness, and uniformity by a simple and facile method, because it is not easy to find an appropriate mask in nanometer scales with a high etch selectivity over the resist. Due to the difficulties in obtaining the ultrahigh selectivity of the SiO_2_ layer to PR, new mask schemes have been developed for nanoscale patterning, such as using multilayer structures, which are typically composed of a SiO_2_, SiN or SiON hard mask, an amorphous carbon layer (ACL), and SiO_2_ underlayer [19, 20]. The final SiO_2_ underlayer is patterned with an etched ACL pattern. This kind of multilayer stacks can improve the etch selectivity of PR to substrate materials during plasma etching. However, the multilayer mask plasma etch involves usually a complex process requiring expensive machinery and a very high process development cost. In addition, good control of the etch selectivity between adjacent layers in the structures is important for next layer etch process. However, these techniques are extremely expensive, complex, and accessible only to large-scale integrated circuit manufacturers. Any simplification of these processes offers a great advantage in both efficiency and cost, particularly for relatively small-scale fabrication typical in research and scientific fields.

In addition to top-down methods presented above, a typical bottom-up method such as self-assembly of block copolymer has drawn considerable attention for the fabrication of highly ordered nanostructures in recent years, since it can access extremely dense and complex nanostructures over a large area for device applications [21, 22]. The method is inherently a bottom-up patterning strategy capable for fabrication of nanostructures in a cost-effective and high throughput way over a large area but usually suffers from low structural regularity and worse uniformity. In recent years, some highly ordered nanostructures of Si and SiO_2_ have been successfully fabricated with this technology [23, 24]. However, there are some challenges to restrict the patterning transfer of block copolymer to various substrate materials. Among of them, etch resistance of block copolymer is inherently not strong enough as an etch mask to produce desirable nanostructures during pattern transfer. Alternatively, an intermediate mask layer, such as Cr, is often used to serve as an etch mask to improve etch resistance of the block copolymer in order to fabricate a wide variety of different nanostructures [25, 26]. Although the nanofeatures can be obtained by combing with a lift-off process, it is very difficult to completely remove all Cr metal film around the pattern edges because of molecular interaction forces. This will result in severe edge roughness, which can be easily subsequently transferred into the underlying substrate materials. In addition, the risk of metal contamination makes the self-assembly difficult to apply to currently standard process, limiting mass production and readily convenient integration into practical CMOS devices. Therefore, the technique is now not likely to be integrated into traditional semiconductor industry.

Apart from metal Cr, inorganic oxide alumina (Al_2_O_3_) is also introduced as an etch mask to fabricate silicon-based nanostructures [27, 28]. The material demonstrates a fairly high etch resistance over silicon and SiO_2_ capable of fabricating various silicon-based nanostructures. However, a significant drawback is that aluminum fluoride (AlF_x_) film will be easily formed on the etched sidewalls and wafer surfaces as soon as the Al_2_O_3_ material is exposed to fluorine-based plasmas. The formation and accumulation of nonvolatile AlF_x_ layer is known to be a serious issue for plasma etching processes: it causes process drifts (generating changes in etch rate, etching profile, selectivity, or uniformity) and metallic contamination of wafers [29, 30]. It is especially worthwhile noting that AlF_x_ material is extremely etch resistant, and it cannot be removed from sidewalls clearly and requires special cleaning chemistries or strategies. Furthermore, the deposition of the etch products on the sidewalls and wafer surfaces may modify the surface reaction probability of the reactive species of the plasma, which in turn modifies the plasma chemistry and process performances [31]. Indeed, it is considered to be critical to result in a bad process instability and reproducibility with worse process controllability.

The pattern transfer routes presented above are generally complicated or incompatible with currently available semiconductor equipment and process. Consequently, with rapid progress of nanotechnology, there is an increasing demand for innovation in nanostructure fabrication techniques by a simple and efficient approach using readily available nanofabrication tool but with a capability for fabricating highly controlled silicon-based nanostructures.

In this paper, we present an attractive and innovative fabrication technique of silicon-based nanostructures over a large area by using amorphous silicon (α-Si) material, which is deposited or thermally grown on a dielectrics substrate such as silicon oxide (or silicon nitride). Actually, it is well known that this material has attracted considerable interest of a large number of scientists and researchers, who, over the years, have used it in a wide variety of applications especially in energy storage devices from photovoltaics to thin film transistors (TFTs) in flat panel displays. In all of these cases, one most attractive point is that α-Si can be deposited very easily at moderate temperatures with a low thermal budget by a few different methods, such as plasma-enhanced chemical vapor deposition or thermally growth technique. These fabrication methods will not pose any impact at all on the other layers on the silicon substrate.

However, to the best of our knowledge, although α-Si material has obtained a wide application in semiconductor industry over past years, it has not been deeply investigated yet as hard mask for the fabrication of silicon-based nanostructures until now. The key point of the approach is that a highly tuned etch selectivity of SiO_2_ over α-Si material can be easily obtained from low to even infinitely high values by optimized process conditions, which has been revealed according to previous studies by us [32]. At the same time, we have reported α-Si material used as a robust capping layer that can generate successfully sub-30-nm gate patterns with smooth and perfectly vertical sidewalls for advanced 22-nm and 14-nm nodes CMOS devices, respectively [32, 33].

Here, based on our previous work, the α-Si material will be used as a versatile mask layer to fabricate a wide variety of periodic SiO_2_ nanostructures including SiO_2_ nanotrench, Si nanoline, and nanofin to aim at broadening application fields of the material by a combination with electron beam (e-beam) lithography. In addition, we will make further investigation to explore well-controlled processes for the fabrication of sub-10-nm silicon nanostructures. Compared with other fabrication techniques reported before, the novel approach is simple, efficient, and easy to implement. It will be expected a new emerging and disruptive technology with low process complexity and good compatibility with existing silicon integrated circuit and is an important step towards facile realization of cost-effective nanoelectronic and optoelectronic devices.

## Methods

### Wafer Preparation

All experiments were conducted on 200-mm single crystal silicon substrates (p-type, (100), 1–10 Ω·cm). First, SiO_2_ film was deposited over the bulk silicon substrate using plasma-enhanced chemical vapor deposition (PECVD) followed by α-Si material thermally grown over the SiO_2_ layer using a rapid thermal processing (RTP) tool. Then, the electron beam resist using a negative tone resist AR-N-7520 (non-chemically amplified resist) was spin-coated on the underlying α-Si material by Kingsemi automatic track with a post-applied baking temperature of 180 °C for 180 s. To obtain highly dense nanoline and nanotrench arrays, the resist exposure was performed at a Gaussian (spot) beam system, NBL NB5. After e-beam writing, post-exposure baking (PEB) of 75 °C for 120 s was performed. The wafers were developed of 60 s in 2.38 % TMAH (tetramethylammonium hydroxide) developer and then rinsed with DI water. Post-development baking (PDB) of 130 °C for 120 s for further drying and hardening of e-beam resist was applied.

### Fabrication of SiO_2_ Nanostructures

**Fig. 1:**
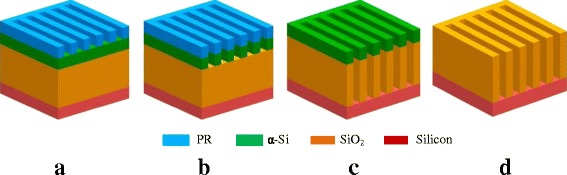
Schematics of the process for fabricating SiO_2_ nanostructures. **a** E-beam lithography. **b** α-Si mask is opened using the resist mask by RIE. **c** SiO_2_ nanofeatures are produced by RIE. **d** α-Si mask is selectively removed using wet etch in TMAH solution to form final SiO_2_ nanostructures

Figure 1 shows a schematic illustration of the process for fabricating SiO_2_ trench nanostructures. A reactive ion etch (RIE) process is used to transfer exposed resist patterns into the underlying α-Si film by an inductively coupled plasma etch tool (Lam TCP 9400DFM) using Cl_2_/HBr/O_2_ plasma chemistries. It can provide a sufficiently high selectivity between α-Si material and the resist because etch property of α-Si material is inherently similar to that of poly-silicon. It is well known that poly-silicon etch has been applied in semiconductor fabrication for many years. Moreover, it must be pointed out that the e-beam resist is very necessarily removed after α-Si patterning, which can be easily achieved by an O_2_ plasma strip in combination with a wet cleaning process in dilute hydrofluoric acid (DHF) followed by sulfuric peroxide mixtures (SPM). This is a critical step to achieve good uniformity and smooth sidewalls for subsequent SiO_2_ nanopatterning because the resulting etched pattern edge roughness and sidewall surface will be easily impacted by some residues occurred during α-Si etch. Otherwise, it will be very difficult to obtain desirable SiO_2_ nanostructures with highly smooth sidewalls.

In the following, the critical SiO_2_ layer can be patterned by Lam Exelan HPT tool with fluorocarbon-based plasma chemistry. It is worthwhile noting that different plasma mixture gases can be used for different SiO_2_ nanostructures to meet different stringent requirement from a wide variety of specific applications in nanofabrication. In this work, we have demonstrated two different SiO_2_ nanostructures including nanotrench and nanoline. For the former, to maintain good profile control, heavily polymerizing plasma chemistries, such as C_4_F_6_/CO/Ar/O_2_, are often used by depositing a thin passivation layer on the exposed surfaces. However, the latter often uses relatively lean polymerizing plasma chemistries, such as CF_4_/CHF_3_/Ar/O_2_, CF_4_/CH_2_F_2_/Ar/O_2_, in order to keep nearly vertical etch profile and smooth sidewalls. Their aims are mainly meet the requirement from specific nanostructure applications. Then, the remaining α-Si mask can be selectively removed by wet etch in TMAH solution without any damage on underlying substrate layer.

### Fabrication of Si Nanostructures

Based on SiO_2_ nanostructures fabricated above, Si nanostructures can be easily produced by a RIE process. Obviously, silicon substrate etch is very similar to α-Si etch with facile fabrication characteristics. Especially, the remaining α-Si material after etch previously can be completely removed during silicon patterning without requirement of an extra process and no significant pattern damage.

## Results and Discussion

### Fabrication of SiO_2_ Nanostructures

To demonstrate the important applications of our novel method, a facile process fabricating SiO_2_ trench nanostructures with below 45 nm will be firstly developed using α-Si mask, as shown in Fig. 1 showing a schematic illustration. Here, the e-beam dose is 500 μC/cm^2^ for the exposure of nanopatterns, performed at an acceleration voltage of 80 kV with a beam current of 3 nA with small beam spot size. The critical dimension of the resist arrays patterned using the e-beam lithography is measured to be about 40-nm width with a period (pitch) of 80 nm. The etch process for the α-Si mask is highly suggested using a mixture of halogen-based not SF_6_-based plasma chemistries, because the latter will easily induce a severely deteriorated pattern edge roughness due to weak resistance of the resist to fluorine radical^16^.

**Fig. 2:**
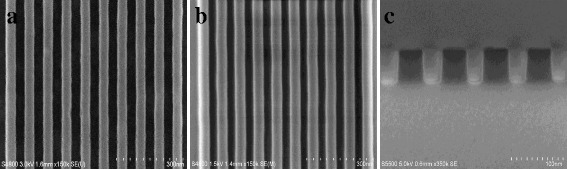
SEM images of α-Si mask opening with 40-nm line width and 40-nm spacing. **a** The resist is patterned by e-beam lithography, and the bright area is the line. **b** α-Si nanofeatures are produced by a RIE process in ICP etcher by Cl_2_/HBr/O_2_ plasma chemistry. **c** Cross-sectional view of **b** shows a highly vertical and smooth etch profile with a superior etch uniformity

As shown in Fig. 2, periodic top-down and cross-sectional SEM images of α-Si patterns have been successfully fabricated using the e-beam resist as a mask, whereby the resist is still remained demonstrating a sufficiently high etch selectivity between α-Si and resist, such resulting in a good pattern transfer with a high fidgety. It can be clearly seen that 50-nm thick highly ordered α-Si patterns show very smooth sidewalls by using optimized process conditions. In addition, the resulting etched α-Si mask demonstrates an almost vertical profile with only slightly tapered sidewall angle. A detail of process condition can be described as follows: 10 mtorr chamber pressure, 250 W source power, −300 V bias voltage, 200 sccm HBr, and 1 sccm O_2_ flow rate. In most of applications, at least 30 % over etch is necessarily required to make sure a complete opening of α-Si mask layer in order to improve image quality of subsequent nanopatterns transfer.

**Fig. 3:**
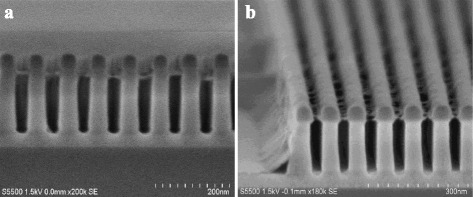
SEM views of SiO_2_ nanostructures with top CD of around 35 nm. **a** Highly passivated polymer films deposited on the trench sidewalls. **b** Tilted view of **a** showing residual polymers on the trench sidewalls and wafer surfaces

Then, α-Si patterns produced above can be directly served as an etch mask to feature various designed SiO_2_ nanostructures by appropriate pattern transfer processes. Figure 3 shows a top-down and cross-sectional SEM images of SiO_2_ nanotrench arrays on the silicon substrate. Note that, here, it should be pointed out that the remaining resist film after α-Si patterning is very necessarily removed in order to avoid a potential impact on the SiO_2_ layer etch process. Otherwise, in this case, it will be very difficult to obtain smooth and desirable SiO_2_ features, and a severe pattern distortion is very likely produced. The resist removal can be achieved by an O_2_ plasma strip in combination with a wet cleaning process in dilute hydrofluoric acid (DHF) followed by sulfuric peroxide mixtures (SPM). Then, the highly ordered α-Si patterns formed previously are transferred into the underlying SiO_2_ film by LAM Exelan Hpt etcher.

Actually, pattern transfer with a good fidelity into the underlying SiO_2_ film to create a high aspect ratio structure is always a challenge in nanometer scales. To maintain accurate profile control to SiO_2_ layer, a standard patterning strategy in the industry is to use heavily polymerizing plasma chemistries to form a thin fluorocarbon polymer layer on all exposed surfaces during the etch process.

As shown in Fig. 3, the etched trench shows a slightly tapered profile which will be more favorable for subsequent film filling. Especially, the α-Si layer is almost completely kept showing a superior resistance to the SiO_2_ material by fluorocarbon base plasma chemistries using a gas mixture of C_4_F_6_ 7 sccm, CO 90 sccm, O_2_ 5 sccm, and Ar 150 sccm at a chamber pressure of 65 mtorr with a source power of 1600 W and a bias power of 1300 W. In this step, the etch process not only can suppress the etch stop with processing but also can assure a desirable etch profile to be achieved and does not cause any pattern distortion, which is very significant to nanoelectrionic devices application.

It is well known that RIE process generally induces surface modifications such as nonvolatile fluorocarbon polymers on the exposed surfaces. These highly passivated polymers contribute to easy formation of a high resistance material which increases the contact resistance significantly and thus also raises device reliability concerns. For these reasons, these polymers must be selectively and completely removed before subsequent film filling inside the trench to avoid contamination and achieve good adhesion and coverage. As a consequence, their successful removal is very critical for subsequent device integration. However, it is well known that these polymers are nonvolatile and also chemically and thermally stable due to their inherently organic characteristics. Moreover, fundamentally, it is very difficult to remove such polymers completely in high aspect ratio trench nanostructures especially in nanometer scale size. Here, it can be easily seen from Fig. 3 that, although etched SiO_2_ nanostructures have been performed a typical cleaning after etch, the resulting etch result still shows a fairly rough surface characteristic due to difficulties in remove and dissolve chemically these polymers on the trench sidewalls. It is evidently seen that a large number of polymer films are still left on the wafer surfaces and trench sidewall surfaces, especially in outer side of trenches, as shown in Fig. 3b. It indicates that current post-RIE cleaning method is not very effective in removing fluorocarbon polymer films. The post-RIE method is based on a typical ex situ O_2_ plasma strip in combination with a wet clean process which includes a dip in dilute hydrofluoric acid (DHF) followed by sulfuric peroxide mixtures (SPM) and ammonia peroxide mixtures (SC1) chemicals.

Although, a large number of studies have been carried out on the dry cleaning of RIE induced polymers in this aspect, most of studies focus on the ex situ O_2_ plasma removal of polymers, because it is thought to be the most effective method. It is achieved by an oxidation reaction with the polymers followed by a chemical wet clean, which can easily complete the cleaning process to avoid leaving polymers behind [34, 35]. However, as the feature size gets smaller to nanometer scale, the typical process has been found to be less effective in removing organic polymers completely.

**Fig. 4:**
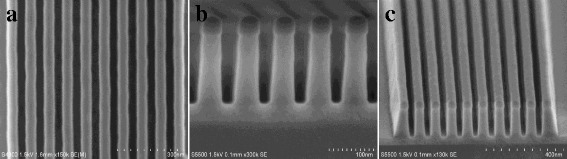
Top-down and cross-sectional SEM views of SiO_2_ trench nanostructures using newly developed cleaning process. **a** The SiO_2_ trench arrays fabricated show highly uniform and smooth characteristics on whole wafer surfaces. **b** Passivated films deposited on the trench sidewalls have been completely cleaned using the novel in situ plasma treatment. **c** Tilted view of **b** shows highly aligned and uniform arrays

In this study, we propose an in situ dry cleaning approach by an immediate O_2_ plasma strip after SiO_2_ etch in the reaction chamber. It proves to be very effective in organic polymer removal because oxygen plasma attacks the fluorocarbon film at unsaturated bonds and forms volatile products; thus, the thick passivation film deposited on the surface can be oxidized and broken into smaller fragments by the in situ O_2_ plasma treatment. Then, a typical wet clean process can be followed to remove those residual fragments as stated above. As shown in Fig. 4, it indicates very clean and smooth sidewall surfaces with a top width of around 35 nm. In addition, all etched SiO_2_ trenches are highly uniform. The etched depth of the SiO_2_ trench nanostructures can be easily controlled by processing time.

**Fig. 5:**
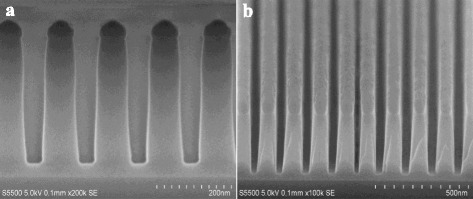
Cross-sectional SEM views of SiO_2_ nanostructures with top CD of 70 nm. **a** Smooth trench sidewalls with a high aspect ratio of 5:1. **b** Tilted views of **a** shows highly aligned and uniform arrays

With the novel technique, α-Si has demonstrated a great potential for the fabrication of SiO_2_ nanostructures with smooth sidewalls. The facile fabrication approach can be easily extended to produce a wide variety of nanostructures with smaller or larger sizes. Although e-beam lithography is the most commonly used technique in constructing nanostructures with high degree of control, the generation of secondary electrons during electron bombardment makes it difficult to pattern with sub-10-nm resolution. However, our approach is not intrinsically limited to the dimensions, densities, and shapes, since successful realization of these arrays mainly relies on a superior etch resistance of SiO_2_ over α-Si material. In this case, we provide another demonstration that indicates highly ordered periodic SiO_2_ nanotrench arrays with a top width of around 70 nm, as shown in Fig. 5.

### Fabrication of Silicon Nanolines

**Fig. 6:**
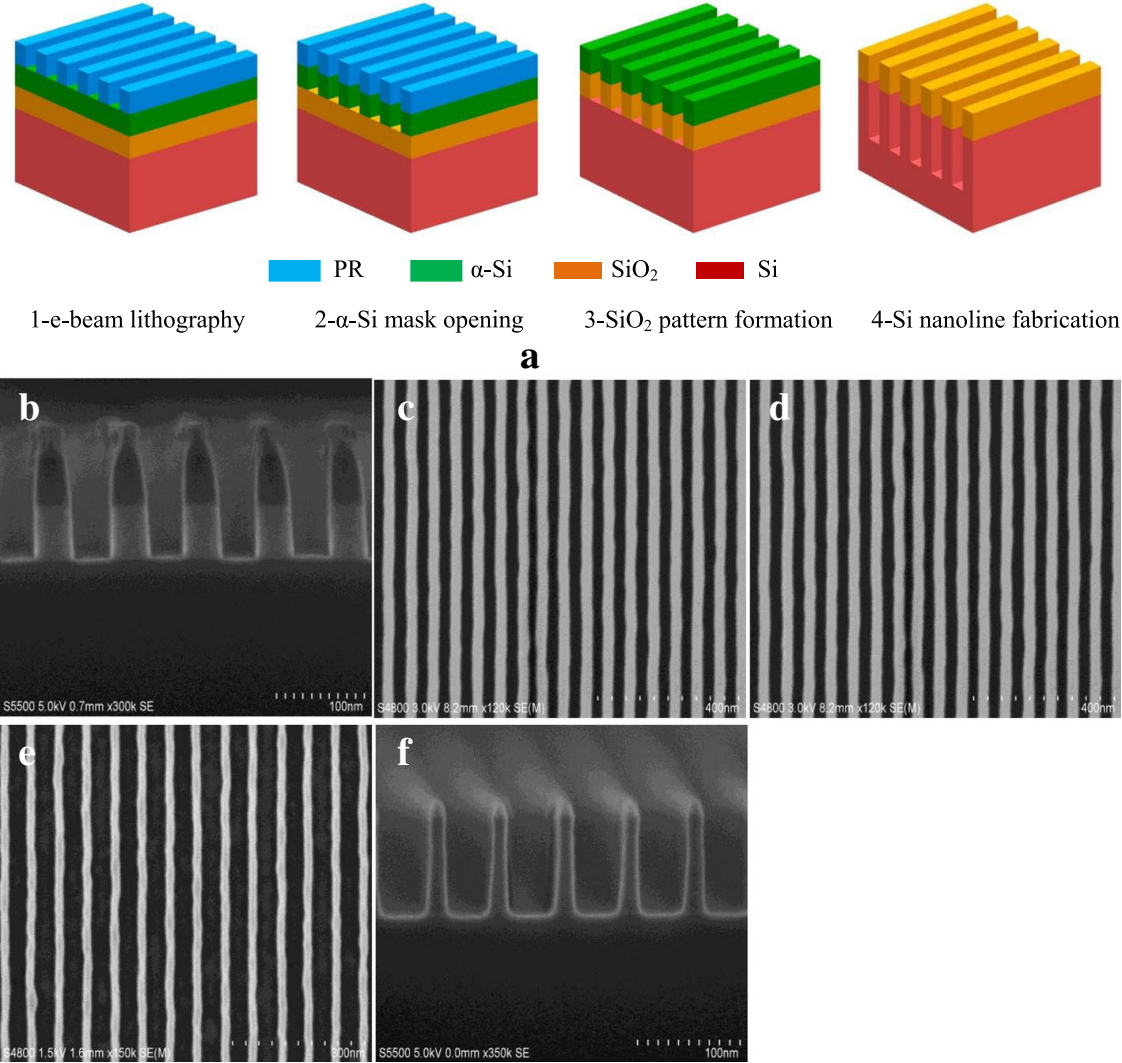
Fabrication of highly controlled periodic silicon nanoline arrays. **a** A schematic illustration for the fabrication of silicon nanoline structures. **b** Cross-sectional views of etched SiO_2_ nanolines using α-Si mask. **c**–**e** Top-down SEM images of parallel silicon nanoline arrays with smooth sidewalls showing 40-, 30-, and 20-nm CD, respectively. **f** Cross-sectional SEM images of **e** with a high aspect ratio of 5:1

The nanostructures we have generated suggest that the simple, top-down patterning technique can achieve sub-40-nm features. In addition to the fabrication of SiO_2_ nanostructures, we can easily extend this method to generate a large variety of silicon nanostructures. For example, large area silicon nanostructures can be facilely obtained by transferring the SiO_2_ nanopatterns fabricated previously into silicon substrates to produce desirable arrays and replicate the nanostructures using a RIE process. As shown in Fig. 6a, the resulting etched SiO_2_ nanoline arrays, with a width of 40 nm, a period (pitch) of 80 nm, and a height of 50 nm, have been successfully demonstrated using plasma mixture gases consisting of CF_4_ and CHF_3_. Smooth and nearly vertical etched sidewalls show an almost perfect pattern transfer from the α-Si mask. After patterning SiO_2_, the top α-Si mask layer is not damaged or attacked at all in vertical direction except a slight lateral loss at the top of the layer. It implies a relatively high etch selectivity between them. In this case, it is obviously expected that SiO_2_ nanoline arrays with a smaller size and period may be achieved by the facile fabrication approach combined with a more aggressive lithography technology such as self-assembly of block copolymer.

In addition, the SiO_2_ nanopatterns formed above can be directly used as an etch mask to transfer into the underlying various electronic function materials or layers such as silicon, germanium, SiGe, transistor gate, III–V materials, or other semiconductor materials to fabricate specifically required nanostructures by typical RIE processes, as shown in Fig. 6a. In the following, we give an exemplification to show a typical fabrication of silicon nanoline arrays applied in such as photonic crystal or biosensor devices. Figure 6b shows etched SiO_2_ nanopatterns with a nearly vertical sidewall profile using a mixture gas of CF_4_ and CHF_3_. Then, it can be precisely transferred into the underlying silicon material to produce highly controlled silicon nanoline arrays with 40-nm width and 40-nm spacing, as shown in Fig. 6c. Similarly, silicon nanoline arrays with various different sizes can be controllably fabricated by the approach, as presented in Fig. 6d, e with 30-nm and 20-nm width, respectively.

The simple method shows a high scalability and superior compatibility with standard top-down nanofabrication. Here, it needs to mention that, due to resolution limit of e-beam resist, silicon nanoline arrays with a minimum width of 20 nm and a period of 60 nm is only shown. It indicates a fairly high aspect ratio structure of near 5:1 with almost smooth sidewalls, which proves again that the ultimate size limit of nanostructures fabricated is determined by the patterning capability of lithography technology rather than the approach itself proposed in this work. Especially, it is worthwhile noting that the remaining top α-Si after SiO_2_ patterning has been completely removed during the fabrication of silicon nanostructures, because α-Si material behaves a similar etch property to silicon. As shown in Fig. 6f, no top α-Si remaining can be observed, which significantly avoids the use of an extra process, demonstrating an enhanced process window.

### Fabrication of Silicon Nanofins

**Fig. 7:**
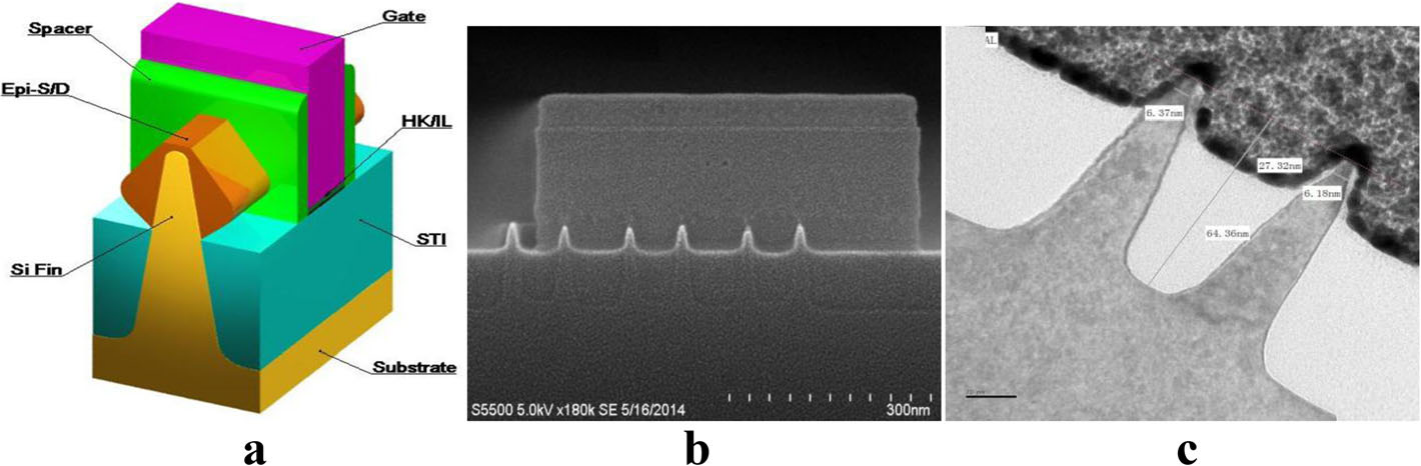
Fabrication of highly aligned silicon nanofin arrays. **a** The three-dimensional view of bulk FinFET device. **b** Cross-sectional SEM views of nanofin arrays. **c** High-resolution TEM image of Si nanofins with top CD around 5 nm through a trimming treatment

In this work, due to the limit of our e-beam lithography as stated above, it is very hard to pattern silicon nanostructures with sub-10-nm resolution. However, by combing with other assisted post-treatment methods such as oxidation or anneal processes, fabricated silicon nanostructures can be further trimmed to produce size controllable nanostructures with smaller lateral resolution than 10 nm. For example, for 14-nm node FinFET devices, top CD of fin arrays is generally less than 10 nm, which can be easily fabricated by above methods. The three-dimensional view of bulk FinFET device is shown in Fig. 7. Note that, here, we only demonstrate a cross-sectional view parallel to bulk fin. Detailed fabrication process flow of bulk FinFET device is referred to our previously published paper [32]. it is clearly observed that highly aligned silicon nanofin arrays are significantly trimmed with top CD of around 5 nm by pattern transfer of α-Si/SiO_2_ masks followed by a dry oxidation treatment. This fully demonstrates a great potential of α-Si material in novel device fabrication.

## Conclusions

We have proposed a novel facile nanofabrication technique of highly controlled silicon-based nanostructures using a simple α-Si material. It is a fully CMOS-compatible new patterning strategy to transfer the resist pattern into the underlying substrate layer. Our results demonstrate that the novel approach has own excellent advantages compared with other nanofabrication techniques described thus far. First, the novel nanofabrication leads to efficient and easy formation of silicon-based nanostructures with high process controllability over a large area. Second, the novel technique can be applied to pattern most materials or layers in IC fabrication such as silicon, germanium and transistor gate, or dielectric materials to fabricate specifically required nanostructures by RIE processes. Finally, we believe that smaller nanostructures may be achievable by using appropriate templates, such as a combination with DSA. As a result, we believe that the novel nanofabrication will serve as an excellent alterative for creating a wide variety of silicon-based nanostructures with high resolution and process controllability.
